# Serum soluble T cell immunoglobulin mucin domain-3 as an early predictive marker for severity of acute pancreatitis; a retrospective analysis

**DOI:** 10.1186/s12876-022-02537-x

**Published:** 2022-12-16

**Authors:** Fushuang Wang, Minghui Zhu, Yao Meng, Min Lin

**Affiliations:** 1 Department of Gastroenterology, The Affiliated Changzhou No.2 People’s Hospital of Nanjing Medical University, No.188 Gehu Road, Wujin District, Changzhou, Jiangsu Province, China; 2 Dalian Medical University, No.9 of Lushun South Road, Dalian, Liaoning Province China; 3 Department of Gastroenterology, The Third Affiliated Hospital of Soochow University, No.185 Juqian Road, Tianning District, Changzhou, Jiangsu Province China

**Keywords:** Soluble TIM-3, Acute pancreatitis, Severity, Prediction, Scoring system

## Abstract

**Background:**

Early prediction of severe acute pancreatitis (SAP) plays an important role in timely treatment decisions. Soluble T cell immunoglobulin and mucin domain-3 (sTIM-3) has been applied as a potential biomarker for the prediction of many diseases, while its predictive ability for AP severity remains largely unexplored. In this study, we aimed to identify whether serum sTIM-3 could be used as an indicator of AP severity in the early stage of the disease.

**Methods:**

A retrospective study was conducted. The enrolled AP patients should meet the 2012 Atlanta guideline and have an onset to admission ≤ 48 h.

**Results:**

A total of 94 AP patients were enrolled in the current analysis, including 42 (45%), 35 (37%), and 17 (18%) patients were diagnosed as mild AP (MAP), moderately SAP (MSAP), and SAP, respectively. SAP patients had significantly higher the white blood cells (WBCs) count, red blood cells (RBCs) count, C-reactive protein (CRP) level, direct bilirubin level, creatinine and procalcitonin levels compared with MAP and MSAP patients. Among SAP and MSAP patients, significantly higher APACHE II, BISAP, and MCTSI scores were observed compared with MAP patients, and there was significant difference in APACHE II and BISAP scores between SAP and MSAP patients. Stepwise multivariate linear regression analysis showed that the concentrations of serum sTIM-3, as well as the BISAP and MCTSI scores, were significantly associated with the severity of AP. The areas under the ROC curve were 0.914 (95% CI, 0.865-0.963), 0.855 (95%CI, 0.742-0.968) 0.853 (95%CI, 0.768-0.938), and 0.746 (95%CI, 0.633-0.860) for BISAP score, APACHE II score, sTIM-3 level, and MCTSI score, respectively.

**Conclusions:**

Serum sTIM-3 might be ultimately incorporated into a predictive system for assessing the severity of AP.

**Supplementary Information:**

The online version contains supplementary material available at 10.1186/s12876-022-02537-x.

## Introduction

As an acute inflammation of the pancreas, acute pancreatitis (AP) is clinically characterized by abrupt onset of deep epigastric pain and biochemically diagnosed by an increase in serum amylase or lipase. Approximately 70-80% of AP patients have a mild disease course. However, in 20-30% of patients [1], it develops to a severe course with a mortality rate of 15-20% [2]. The pathogenesis of AP is still an enigma to clinicians and basic research scientists.

Most severe acute pancreatitis (SAP) patients develop systemic inflammatory response syndrome (SIRS) in the early stage of the disease. SIRS is recognized as one of the most important indicators for the occurrence of persistent organ failure, which is responsible for morbidity and mortality in most SAP patients [3]. Furthermore, the early prediction for SAP is important for treatment decisions.

In recent years, with rapid advances in diagnostic tests, studies on more biomarkers for AP have received extensive attention. As a surface molecule expressed on immune cells, T cell immunoglobulin and mucin domain-3 (TIM-3) plays a fundamental role in immune regulation. Elevated soluble TIM-3 (sTIM-3) may serve as a potential biomarker for the prediction of the disease activity of sepsis [4], systemic lupus erythematosus (SLE) [5], adultonset Still’s disease [6], autoimmune hepatitis [7] and COVID-19 [8, 9].

Our previous study has shown that serum sTIM-3 participates in the early progression of AP by positively regulating the pro-inflammatory cytokines [10]. In the current study, we aimed to assess whether serum sTIM-3 could represent an early indicator of the severity of AP. Moreover, we measured and compared the levels of serum sTIM-3 in AP patients within 24 h of admission.

## Materials and methods

This retrospective study was approved by the ethical committee of the Changzhou No. 2 People’s Hospital Affiliated to Nanjing Medical University (No. [2022] KY 116-01) and the experiments complied with the Helsinki declaration.

### Study populations

Consecutive patients with AP admitted at our hospital between September 2020 and July 2021 were enrolled in the present study. Diagnostic criteria included two or more of the following characteristics: (1) abdominal pain consistent with AP; (2) serum amylase and/or lipase three times higher compared with the normal upper limit; and/or (3) computed tomography (CT) findings of AP. According to the Atlanta classification, AP was classified into three degrees of severity: mild AP (MAP), moderately SAP (MSAP), and SAP [11]. Exclusion criteria were set as follows: time from pain onset to admission > 48 h, age < 18 years, diagnosis of chronic pancreatitis, history of tumor or immune-related disease.

### Data Collection

This study was a retrospective analysis. General information, including gender, age, body mass index (BMI), pre-existing comorbidities (diabetes mellitus, hypertension, and hyperlipidemia), and substance abuse (alcohol and tobacco) were collected from the medical chart of AP patients. On admission, vital sign (temperature, heart rate, pulse oxygen saturation, blood pressure) were recorded. Blood samples were obtained from AP patients for blood tests within 24 h of admission. Serological tests included serum concentrations of white blood cells (WBCs), red blood cells (RBCs), platelet, C-reactive protein (CRP), aspartate and alanine aminotransferases (AST and ALT), bilirubin, urea, creatinine, procalcitonin (PCT), and serum activities of amylase. The routine tests were conducted on the day of blood collection in the Central Laboratory of Changzhou No. 2 People’s Hospital using automatic analyzers and standard protocols.

Human serum sTIM-3 enzyme-linked immunosorbent assay (ELISA) kits (Shanghai Zeye Biotechnology Co. LTD, China) were used to detect the levels of sTIM-3 according to the manufacturer’s instructions. Briefly, the standard was reconstituted into different concentrations using distilled water, the standard concentrations were considered as the horizontal axis, and the optical density (OD) values were used as the vertical axis. Regressed data were used to create a standard curve using computer software. OD was detected at the wavelength of 450 nm using an xMark microplate reader, and the concentrations of sTIM-3 were calculated according to the standard curve. Serum inflammatory cytokine interleukin-6 (IL-6), interleukin-10 (IL-10) levels were determined using the corresponding commercial ELISA kits (Jiangsu Meibiao Biotechnology Co. LTD, China) according to the manufacturer’s instructions.

Various severity scoring systems, including acute physiology and chronic health evaluation II (APACHE II) score, bedside index for severity in acute pancreatitis (BISAP) score, and modified computed tomography severity index (MCTSI) score, were made during the first 24 h.

Patients were followed until discharge from hospital and the length of stay in the hospital was recorded. They were managed according standard clinical practice.

### Statistical analyses

Statistical analysis was performed using SPSS 22.0 software (IBM, Armonk, NY, USA) and R software (http://www.R-project.org). The measurement data was expressed by mean and standard deviation (xˉ ± s), and the measurement data with skewed distribution was expressed by median (Q1-Q3). The chi-square test (categorical variable), one-way analysis of variance (ANOVA) and Student-Newman-Keuls (SNK) (normally distributed continuous variable), and Kruskal Wallis test (skew continuous variable) were used to analyze the differences between and within groups, respectively. Associations of study variables with acute pancreatitis severity were analyzed using multiple linear regression model. We calculated unadjusted and adjusted estimates using exact methods and asymptotic methods which provided (β) and corresponding 95% confidence intervals (95%CI), respectively. We adjusted for features that, when added to this model, changed the matched by at β value at least 10%. Three regression models were used for analysis in this study: Model 1: unadjusted for covariates. Model 2: adjusted for temperature, pulse oxygen saturation, white blood cell count, CRP, procalcitonin and length of hospital stay. Model 3: adjusted for sTIM-3, IL-6, IL-10 on the basis of adjusted Model 2.

The receiver operating characteristic (ROC) curve was drawn for sTIM-3 and different scoring systems, and compared the diagnostic efficacy and area under the curve (AUC) of sTIM-3, BISAP, APACHE-II, MCTSI scores using the method from DeLong et al. [12]. *P* value < 0.05 was considered statistically significant.

## Results

### Characteristics of Study Population

A total of 94 AP patients were included in the present study, among which 42 (45%), 35 (37%), and 17 (18%) patients were diagnosed with MAP, MSAP, and SAP, respectively. Table 1 shows that MAP, MSAP, and SAP patients did not differ significantly in terms of age, gender, BMI, AP etiology, tobacco use, alcohol use, and pre-existing comorbidities. In contrast, SAP patients had a higher temperature, faster heart rate, higher mean arterial pressure, and lower pulse oxygen saturation (*P* < 0.05) compared with MAP and MSAP patients. In addition, the length of hospital stay was longer in SAP patients.

**Table 1:** Characteristics of the study population

Characteristic	MAP(*n =* 42)	MSAP(*n =* 35)	SAP(*n =* 17)	*P*-value
Age(y)	47.548 ± 14.750	50.657 ± 15.332	47.941 ± 17.185	0.659
Gender				0.194
Male	20 (47.619%)	22 (62.857%)	12 (70.588%)	
Female	22 (52.381%)	13 (37.143%)	5 (29.412%)	
BMI	25.976 ± 3.605	26.283 ± 3.540	28.212 ± 5.435	0.141
Etiology				0.949
Biliary, n (%)	25 (59.524%)	24 (68.571%)	9 (52.941%)	
Hypertriglyceridemia, n (%)	11 (26.190%)	7 (20.000%)	5 (29.412%)	
Alcoholic, n (%)	2 (4.762%)	2 (5.714%)	1 (5.882%)	
Other, n (%)	4 (9.524%)	2 (5.714%)	2 (11.765%)	
Tobacco, n (%)	3 (7.143%)	1 (2.857%)	2 (11.765%)	0.451
Alcohol, n (%)	4 (9.524%)	3 (8.571%)	5 (29.412%)	0.075
Pre-existing comorbidities				
Diabetes mellitus, n (%)	13 (30.952%)	16 (45.714%)	8 (47.059%)	0.323
Hypertension, n (%)	17 (40.476%)	13 (37.143%)	5 (29.412%)	0.728
Vital signs				
Temperature, °C	36.795 ± 0.391	36.823 ± 0.468	37.265 ± 0.885$#	0.008
Heart rate, beats per minute	82.381 ± 13.676	94.000 ± 17.427&	117.471 ± 24.426$#	< 0.001
Pulse oxygen saturation, %	97.429 ± 1.741	96.686 ± 2.654	90.765 ± 10.059$#	< 0.001
Mean arterial pressure, mmHg	98.069 ± 13.111	99.149 ± 18.890	111.059 ± 27.645$#	0.046
Length of hospital stay, days	7.476 ± 2.540	9.086 ± 3.212	17.059 ± 9.959$#	< 0.001

*Abbreviations:MAP* Mild acute pancreatitis, *MSAP* Moderately severe acute pancreatitis, *SAP* Severe acute pancreatitis

*P* < 0.05 was considered statistically significant. ^&^*P* < 0.05, MAP versus MSAP; ^#^ *P* < 0.05, MAP versus SAP; ^$^*P* < 0.05, MSAP versus SAP

As shown in Table 2, on admission, SAP patients had significantly higher the WBC count, RBC count, CRP level, direct bilirubin level, creatinine and procalcitonin levels (*P* < 0.05) compared with MAP and MSAP patients. However, other biochemical variables, such as hematocrit, ALT level, AST level, total bilirubin level, triglyceride level, urea and amylase levels, did not differ significantly among the MAP, MSAP, and SAP patients.

**Table 2:** The results of laboratory tests on admission according to the acute pancreatitis severity

Variable	MAP(*n =* 42)	MSAP(*n =* 35)	SAP(*n =* 17)	*P*-value
White blood cell count, × 10^9^/L	9.830 ± 4.188	12.028 ± 4.320&	16.350 ± 4.527$#	< 0.001
Red blood cell count, × 10^12^/L	4.647 ± 0.593	4.802 ± 0.667	5.374 ± 0.828$#	0.001
Hematocrit, %	43.752 ± 13.922	45.197 ± 17.827	48.635 ± 6.860	0.513
Platelet count, × 10^12^/L	191.881 ± 62.304	225.086 ± 84.156	243.235 ± 82.945#	0.034
CRP, mg/L	54.088 ± 67.037	92.224 ± 75.537	194.541 ± 152.605$#	< 0.001
ALT, U/L	54.333 ± 152.244	93.870 ± 168.934	252.682 ± 602.560#	0.063
AST, U/L	73.595 ± 133.175	86.691 ± 197.879	228.735 ± 600.135	0.168
Total bilirubin, μmol/L	22.000 (14.500, 33.850)	23.400 (18.100, 39.900)	38.000 (15.65, 126.6)	0.196
Direct bilirubin, μmol/L	7.800 (3.075, 12.200)	10.900 (5.900,1 6.200)	28.000 (10.000,103.500)$#	0.001
Triglyceride, mmol/L	4.238 ± 5.253	10.244 ± 28.837	8.136 ± 7.407	0.350
Creatinine, μmol/L	41.064 ± 29.968	51.709 ± 30.791	82.206 ± 43.672$#	< 0.001
Urea, mmol/L	7.514 ± 16.428	4.809 ± 2.258	5.724 ± 2.599	0.563
Procalcitonin, ng/ml	0.188 ± 0.285	1.026 ± 2.305	5.040 ± 11.073$#	0.003
Amylase, U/L	727.219 ± 981.935	924.914 ± 853.368	895.653 ± 790.804	0.602
sTIM-3, pg/ml	911.615 ± 565.535	1462.801 ± 688.925&	2152.698 ± 632.143$#	< 0.001
IL-6, pg/ml	42.902 ± 28.821	119.501 ± 74.666&	323.593 ± 62.933$#	< 0.001
IL-10, pg/ml	1206.080 ± 566.251	964.443 ± 603.482	760.253 ± 200.418#	0.012
APACHE II score, points	3.619 ± 2.230	5.743 ± 3.119&	12.647 ± 8.177$#	< 0.001
BISAP score, points	1.214 ± 0.606	2.400 ± 0.847&	3.529 ± 0.717$#	< 0.001
MCTSI score, points	1.190 ± 0.994	4.057 ± 1.413&	4.235 ± 1.562#	< 0.001

Abbreviations: *MAP* Mild acute pancreatitis, *MSAP* Moderately severe acute pancreatitis, *SAP* Severe acute pancreatitis, *CRP* C-reactive protein, *ALT* Alanine aminotransferases, *AST* Aspartate aminotransferases, *IL-6* Interleukin-6, *IL-10* Interleukin-10, *APACHE II* Acute physiology and chronic health evaluation II, *BISAP* Bedside index for severity in acute pancreatitis, *MCTSI* Modified computed tomography severity index

*P* < 0.05 was considered statistically significant. ^&^*P* < 0.05, MAP versus MSAP; ^#^*P* < 0.05, MAP versus SAP; ^$^*P* < 0.05, MSAP versus SAP

**Fig. 1:**
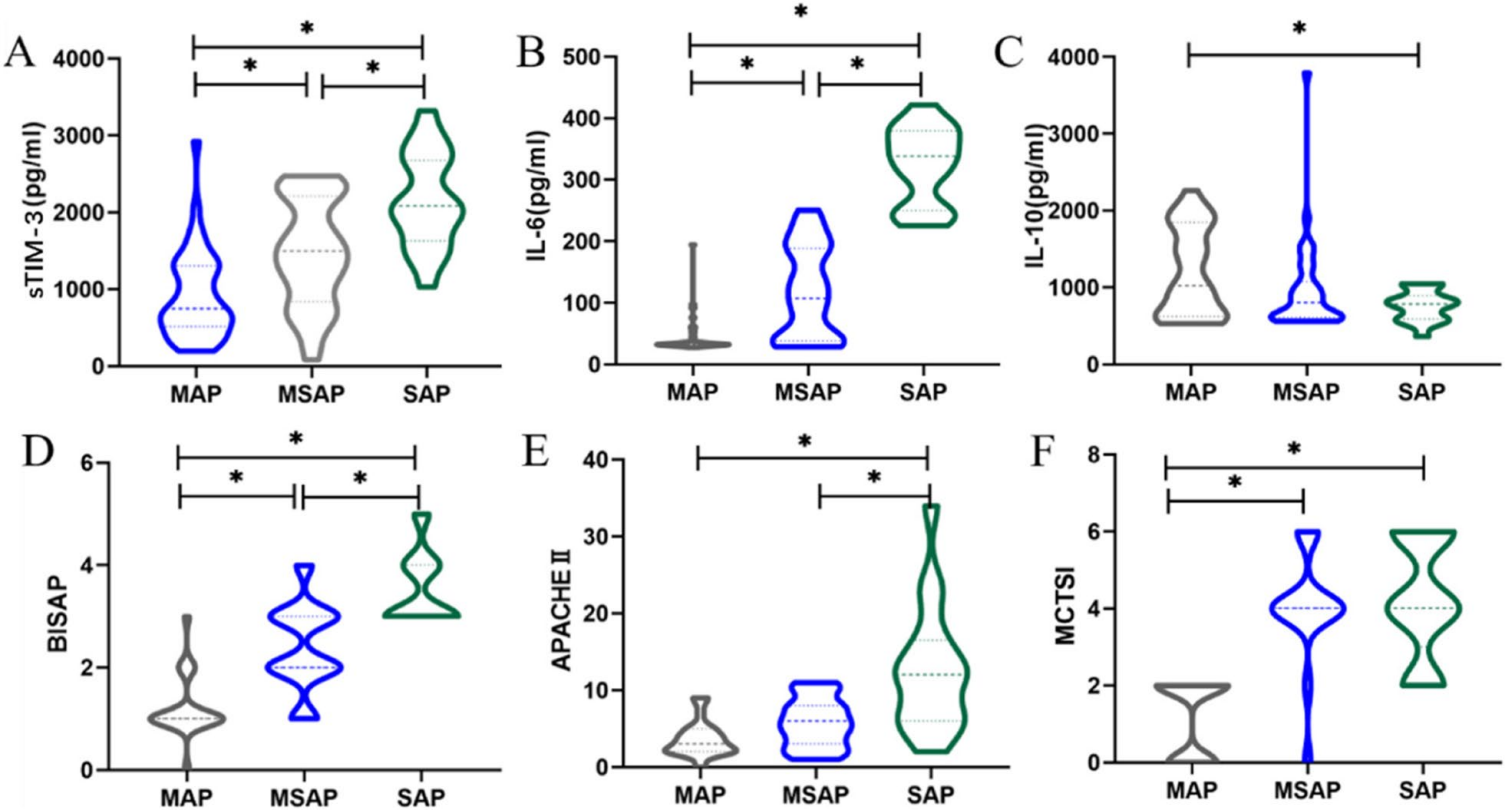
Distribution of sTIM-3, IL-6, IL-10, scoring systems in MAP, MSAP, SAP patients. Abbreviations: The difference in levels of sTIM-3 (**A**), IL-6 (**B**), IL-10 (**C**), BISAP scores (**D**), APACHE II scores (**E**), and MCTSI (**F**) among MAP, MSAP, and SAP patients. *P < 0.05 was considered statistically significant. MAP, mild acute pancreatitis; MSAP, moderately severe acute pancreatitis; SAP, severe acute pancreatitis; sTIM-3, soluble T cell immunoglobulin and mucin domain-3; APACHE II, acute physiology and chronic health evaluation II; BISAP, bedside index for severity in acute pancreatitis; MCTSI, modified computed tomography severity index

In terms of inflammatory cytokines, SAP and MSAP patients had significantly higher IL-6 cytokine level (*P* < 0.05) compared with MAP patients, and there was significant difference in IL-6 cytokine level between SAP and MSAP patients (Table 2, Fig. 1B). While SAP patients had significantly lower IL-10 cytokine level (*P* < 0.05) compared with MAP patients, there was no significant difference in IL-10 level between other two groups (Table 2, Fig. 1C). Among SAP and MSAP patients, significantly higher serum sTIM-3 levels were observed compared with MAP patients, and there was significant difference in sTIM-3 level between SAP and MSAP patients (Table 2, Fig. 1A).

As illustrated in Table 2, Fig. 1D, E, F, among SAP and MSAP patients, significantly higher APACHE II, BISAP, and MCTSI scores were observed compared with MAP patients, and there was significant difference in APACHE II and BISAP scores between SAP and MSAP patients, but there was no significant difference in MCTSI score between SAP and MSAP patients.

### Multivariable linear regression analysis

Univariate linear regression analysis showed that compared with the MAP group, serum sTIM-3 levels in the MSAP and SAP groups increased by 551.186 and 1241.082 pg/mL, respectively, showing significant differences among these groups (Model 1, Table 3). After adjustments for temperature, pulse oxygen saturation, white blood cell count, CRP, procalcitonin, length of hospital stay and IL-6, IL-10(*P* for trend < 0.001) (Model 2,3, Table 3), serum sTIM-3 levels was still gradually increased with the severity of AP. IL-6 cytokine level with the severity of AP was statistically significant, too (Model 1-3, Table 3, *P* for trend < 0.001). IL-10 cytokine level in MAP, MSAP, SAP groups showed a gradual decrease trend (Model 1, *P* for trend =0.012, Model 2, *P* for trend =0.003, Model 3, *P* for trend =0.007, Table 3).

**Table 3:** Univariate and multivariable linear regression analysis for the severity of pancreatitis

	sTIM-3	IL-10	IL-6	BISAP	APACHE-II	MCTSI
	*B (95%CI) P-value*	*B (95%CI) P-value*	*B (95%CI) P-value*	*B (95%CI) P-value*	*B (95%CI) P-value*	*B (95%CI) P-value*
Model 1 (unadjusted)						
MAP	Reference	Reference	Reference	Reference	Reference	Reference
MSAP	551.186 (270.440,831.932) 0.001	−241.637(− 482.203, − 1.070) 0.052	76.599 (51.408,101.790) < 0.001	1.186 (0.861,1.511) < 0.001	2.124 (0.240,4.007) 0.030	2.867 (2.296, 3.438) < 0.001
SAP	1241.082 (888.465,1593.700) < 0.001	− 445.827 (− 747.979, -143.675) 0.005	280.692 (249.052, 312.332) < 0.001	2.315 (1.907, 2.723) < 0.001	9.028 (6.662, 11.394) < 0.001	3.045 (2.328, 3.762) < 0.001
P trend	< 0.001	0.012	< 0.001	< 0.001	< 0.001	< 0.001
Model 2 (adjusted for temperature, pulse oxygen saturation, white blood cell count, CRP, procalcitonin and length of hospital stay)						
MAP	Reference	Reference	Reference	Reference	Reference	Reference
MSAP	477.680 (188.955, 766.405) 0.002	− 281.815(− 537.846, − 25.783) 0.034	70.432 (43.864,97.000) < 0.001	1.024 (0.758, 1.290) < 0.001	1.645(− 0.138, 3.428) 0.074	2.870 (2.271, 3.468) < 0.001
SAP	1188.910 (821.672, 1556.148) < 0.001	−429.235(− 754.890, − 103.581) 0.012	282.954 (249.162, 316.747) < 0.001	2.263 (1.925, 2.601) < 0.001	8.470 (6.202, 10.738) < 0.001	3.049 (2.287, 3.810) < 0.001
P trend	< 0.001	0.003	< 0.001	< 0.001	0.003	< 0.001
Model 3 (adjusted for sTIM-3, IL-6, IL-10 on the basis of adjusted Model 2)						
MAP	Reference	Reference	Reference	Reference	Reference	Reference
MSAP	476.109 (181.573, 770.644) 0.002	− 360.238(− 798.644, 78.169) 0.114	84.528 (53.834,115.222) < 0.001	1.051 (0.779, 1.323) < 0.001	1.123(− 0.171, 2.418) 0.093	2.893 (2.277, 3.510) < 0.001
SAP	819.978 (275.672, 1364.284) 0.004	− 776.503(− 2139.439,586.434) 0.270	297.153 (212.197, 382.110) < 0.001	2.134 (1.631, 2.637) < 0.001	3.715 (1.323, 6.107) 0.003	2.524 (1.384, 3.663) 0.001
P trend	< 0.001	0.007	< 0.001	< 0.001	0.003	< 0.001

Abbreviations: *MAP* Mild acute pancreatitis, *MSAP* Moderately severe acute pancreatitis, *SAP* Severe acute pancreatitis, *sTIM-3* Soluble T cell immunoglobulin and mucin domain-3, *APACHE II* Acute physiology and chronic health evaluation II, *BISAP* Bedside index for severity in acute pancreatitis, *MCTSI* Modified computed tomography severity index

*P <* 0.05 was considered statistically significant

BISAP score and MCTSI score were significantly associated with the severity of AP (Table 3, *P* for trend < 0.001). Multivariate linear regression analysis indicated that the APACHE II score also showed a gradual upward trend, but the increase was not significantly different between MSAP group and MAP group (model 2, *P* = 0.074, model 3, *P* = 0.093, Table 3).

### Predictive value of sTIM-3 and different scoring systems in SAP

**Fig. 2:**
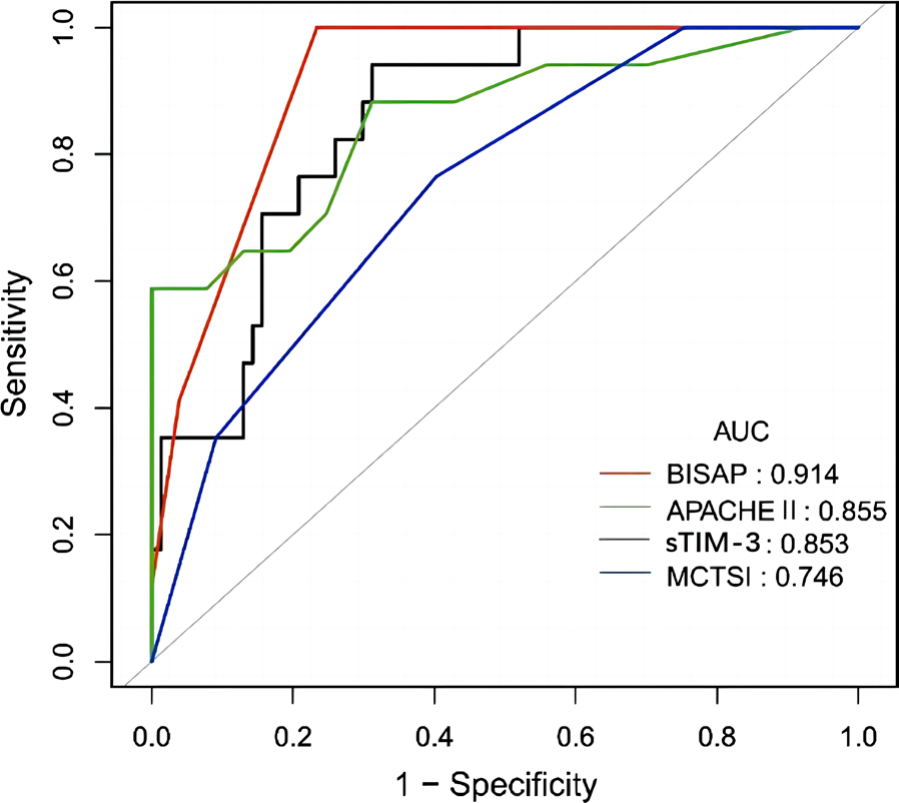
ROC curves for BISAP, APACHE II, MCTSI score, and sTIM-3 in prediction of SAP. Abbreviations: sTIM-3, soluble T cell immunoglobulin and mucin domain-3; APACHE II, acute physiology and chronic health evaluation II; BISAP, bedside index for severity in acute pancreatitis; MCTSI, modified computed tomography severity index; AUC, area under the curve

The ROC curves were used to compare the diagnostic performance of BISAP score, APACHE II score, sTIM-3 level, and MCTSI score. The results showed that the performance of BISAP score and APACHE II score in predicting SAP (AUC was 0.914 and 0.855, respectively) were better than sTIM-3 level (AUC = 0.853), but the difference was not statistically significant (*P* = 0.159 and 0.969, respectively) (Fig. 2, Table 4).

**Table 4:** Diagnostic value for different scoring systems versus sTIM-3 in SAP

Variable	AUC (95% CI)	Cut-off	Specificity (%)	Sensitivity (%)	Accuracy (%)	+LR	-LR	PPV (%)	NPV (%)
sTIM-3	0.853 (0.768-0.938)	1400.247	68.83	94.12	73.4	3.02	0.086	40	98.15
BISAP	0.914 (0.865-0.963)	3	76.62	100	80.85	4.278	0	48.57	100
APACHE-II	0.855 (0.742-0.968)	12	100	58.82	92.55	Inf	0.412	100	91,67
MCTSI	0.746 (0.633-0.860)	4	59.74	76.47	62.77	1.9	0.394	29.55	92

Abbreviations: *sTIM-3* Soluble T cell immunoglobulin and mucin domain-3, *APACHE II* Acute physiology and chronic health evaluation II, *BISAP* Bedside index for severity in acute pancreatitis, *MCTSI* Modified computed tomography severity index, *AUC* Area under the curve, *CI* Confidence interval, *LR* Likelihood ratio, *PPV* Positive predictive value, *NPV* Negative predictive value

The cut-off values of the BISAP score, APACHE II score, sTIM-3 level, and MCTSI score for predicting SAP are listed in Table 4. The specificity, sensitivity, accuracy, pre-test/post-test likelihood ratios, positive predictive values (PPV) and negative predictive values (NPV) for different cut-off values are shown in Table 4.

## Discussion

Rapid and accurate prediction of disease course in the early stage of AP remains a challenge. Because only rapid and accurate prediction is achieved, the subsequent precise and appropriate therapeutic intervention can be determined. That is the reason why the availability of accessible and practical parameters can be a valuable perspective. To the best of our knowledge, this is the first study investigated the predictive potential of serum sTIM-3 to predict the severity of AP within the first 24 h of admission. The data indicated that serum sTIM-3 level was increased with the severity of AP. Additionally, compared with the BISAP, APACHE II, and MCTSI score, the predictive value of sTIM-3 was simpler.

Currently, several scoring systems, such as the BISAP, APACHE II, and MCTSI, have good predictive capabilities for disease severity. The APACHE II scoring system is a reliable prognostic scoring system in AP. It can perform as well as the pancreatitis-specific measures using values obtained within the first 24 h of admission [13]. The APACHE II scoring system is primarily applied in the dynamic evaluation of critical patients, and it consists of three parts: acute physiology, age, and chronic health evaluation. Therefore, the APACHE II measure is complicated and inconvenient to clinical application. The Atlanta criteria recommend a cut-off of> 8 for the APACHE II score for severity prediction [14]. However, recent publications have used a cut-off value ranging from > 6 to > 10 in clinical practice [15]. None of these cut-off values achieve an overall accuracy of more than 75–80%. Our study showed the power of the APACHE II scoring system to predict AP severity, and a cut-off of> 12 for the APACHE II score for severity prediction within the first 24 h of admission. Multivariate linear regression analysis indicated that the APACHE II score also showed a gradual upward trend, but the increase was not significantly different between MSAP group and MAP group.

The BISAP scoring system is initially proposed in 2008 and is composed of five indicators (BUN, impaired mental status, SIRS, age, and pleural effusion) to predict the mortality of AP within 24 h of admission [16]. The primary advantage of the BISAP scoring system is simplicity. There is no need for additional computation. In addition, each of the parameters can be easily obtained early in the course of general hospital admission. BISAP scoring system is easy to use, while meta-analysis display it has low sensitivity for the early prediction of AP severity [17]. The MCTSI scoring system is initially proposed in 2004 based on the CT severity index. It includes assessments of pancreatic inflammation and the area of pancreatic necrosis, as well as extra-pancreatic complications on the initial CT scans. These measures are closely related to outcome measures of AP patients. However, peripancreatic fat necrosis is important criteria in MCTSI scoring system. Early diagnosis of acute pancreatitis by the MCTSI scoring system may be delayed because the early stages of acute pancreatitis are usually not accompanied by peripancreatic fat necrosis [18, 19]. In our current study, we determined the values of the BISAP and MCTSI scoring systems to predict AP severity within the first 24 h of admission. Each scoring system has specific applications and advantages, while they also have limitations.

A few reports have shown that the elevated sTIM-3 level may serve as a potential biomarker for predicting the disease activity and severity. In our previous study, we have firstly reported that sTIM-3 participates in the early progression of AP [10]. Although the function of sTIM-3 has not been clarified, it seems to reflect the severity of AP. In the present study, we found that the concentration of sTIM-3 was significantly different among the MAP, MSAP, and SAP patients. Further multivariate linear regression analysis showed the value of sTIM-3 could predict the AP severity within the first 24 h of admission. After adjustments for factors (model 2), the trend of gradual increase was still statistically significant. In the adjusted model 3, the power of sTIM-3 was reduced, while the *P* value was still statistically significant. The cause might be attributed to the interference with IL-6 cytokine. Currently, serum IL-6 cytokine has been proposed as a valuable prognostic biomarker for the early prediction of AP severity [20, 21]. The other various biomarkers, such as CRP and PCT, have been tested for the early prediction of AP severity, while they have not shown a flawless performance [22]. The combination of these two might be an important approach to improve early predictive sensitivity of AP severity, which requires further investigations. Early detection of the tendency toward SAP and early intervention are important in the treatment and reduction of mortality in AP. The BISAP and MCTSI scoring systems have good predictive capabilities for AP severity. Compared with the BISAP and MCTSI scoring systems, serum sTIM-3 also had a good predictive value.

Additionally, in our current study, it should be noted that among the MAP, MSAP, and SAP patients were not significantly higher in terms of BMI, age, pre-existing comorbidities, triglyceride, hematocrit, and PCT [23–26]. The association between these indicators and the severity of AP is controversial in previous reports.

We selected the study population based on strict inclusion and exclusion criteria. In addition, we used multivariable linear regression models to examine the association between variables and AP severity and estimated two models to exclude confounding factors.

There are also several limitations in our study. This study is a single-center retrospective study. The main limitation is the relatively small sample size, especially those with severe disease. Another limitation is that the enrolled patients had an onset time within 48 h because the course of the disease could change dynamically during the first 3 days, making an urgent need to diagnose disease severity in the early stage of AP. According to the 2012 Atlanta guideline, the early stage of AP is defined as the first week after abdominal pain. Therefore, we only enrolled AP patients within 48 h of onset. Furthermore, we should compare the diagnostic efficiency of serum sTIM-3 and various scoring systems among patients within different times of onset for the prediction of AP.

## Conclusions

In summary, the serum sTIM-3 was an objective, comparable, and more sensitive parameter, which had different levels among MAP, MSAP, and SAP patients. Taken together, serum sTIM-3 might be ultimately incorporated into a predictive system for assessing the severity of AP.

## Supplementary Information

**Additional file 1.** A total of 94 acute pancreatitis (AP) patients were enrolled in the current analysis, including 42 (45%), 35 (37%), and 17 (18%) patients were diagnosed as mild acute pancreatitis (MAP), moderately severe acute pancreatitis (MSAP), and severe acute pancreatitis (SAP). General information, including gender, age, Etiology (Biliary, Hypertriglyceridemia, Alcoholic , and Other), body mass index (BMI), pre-existing comorbidities (diabetes mellitus, hypertension, and high triglyceride), and substance abuse (alcohol and tobacco) and Length of hospital stay were collected from the medical chart of AP patients (0 means no, 1 means yes). On admission, vital sign (temperature, heart rate, Breathe, oxygen saturation, Mean arterial pressure) were recorded. Blood samples were obtained from AP patients for blood tests within 24 h of admission. Serological tests included serum concentrations of white blood cells (WBCs), red blood cells (RBCs), Hematocrit, platelet, C-reactive protein (CRP), aspartate and alanine aminotransferases (AST and ALT), Total and Direct bilirubin (TB and DB), urea, creatinine, procalcitonin (PCT), and serum activities of amylase. Various severity scoring systems, including acute physiology and chronic health evaluation II (APACHE II) score, bedside index for severity in acute pancreatitis (BISAP) score, and modified computed tomography severity index (MCTSI) score, were made during the first 24 h. Soluble T cell immunoglobulin and mucin domain-3 (sTIM-3), Serum inflammatory cytokine interleukin-6 (IL-6), interleukin-10 (IL-10) levels were determined using the corresponding commercial ELISA kits..

## Data Availability

The data provided by this research is the original data, obtained from the database search of Changzhou Second People’s Hospital. The datasets generated and analyzed during the current study are not publicly available due to hospital database limitations, but are available from the respective authors upon reasonable request.
